# Is the cup orientation different in bilateral total hip arthroplasty with right-handed surgeons using posterolateral approach?

**DOI:** 10.1186/s13018-018-0789-y

**Published:** 2018-05-23

**Authors:** Xinggui Song, Ming Ni, Heng Li, Xin Li, Xiang Li, Jun Fu, Jiying Chen

**Affiliations:** 1 Orthopaedic Department, Chinese PLA General Hospital, 28 Fuxing Road, Haidian District, Beijing, People’s Republic of China; 2 Orthopaedic Department, Tianjin Medical University General Hospital Airport Hospital, 85 Dongliu Road, Tianjin Free Trade Zone, Tianjin, People’s Republic of China

**Keywords:** Total hip arthroplasty, Anteversion, Inclination, Surgeon handedness, Safe zone

## Abstract

**Background:**

The impact of surgeon handedness on acetabular cup orientation in total hip arthroplasty (THA) is not well studied. The aim of our study is to investigate the difference of cup orientation in bilateral THA performed by right-handed surgeons using posterolateral approach and which cup could be fitter to Lewinneck’s safe zone.

**Methods:**

The study consisted of 498 patients that underwent bilateral THA by three right-handed surgeons in our hospital. Postoperative acetabular cup anteversion and abduction on an anteroposterior pelvic radiograph were measured by Orthoview software (Orthoview LLC, Jacksonville, Florida). Furthermore, the percentage of cup placement within the safe zone was compared.

**Results:**

The mean anteversion was 25.28 (25.28° ± 7.16°) in left THA and 22.01 (22.01° ± 6.35°) in right THA (*p* < 0.001). The mean abduction was 37.50 (37.50° ± 6.76°) in left THA and 38.59 (38.59° ± 6.84°) in right THA (*p* = 0.011). In the left side, the cup was positioned in Lewinnek’s safe zone in 52% for anteversion, 87% for abduction, and 46% for both anteversion and abduction. But the cup placement within Lewinnek’s safe zone was 71, 88, and 62% in the right side, respectively. There were significant differences in the percentage of acetabular cup placement within the safe zone for anteversion (*p* < 0.001) and for both anteversion and inclination (*p* < 0.001). Dislocation occurred in 7.0% (35/498) of cases in left THA and 3.2% (16/498) in right THA. The percentages of patients experiencing dislocation were significantly different between the two sides (*p* = 0.006).

**Conclusions:**

This current study demonstrated that surgeon handedness is likely to be a contributing factor that affects cup inclination and anteversion in bilateral THA and that the placement of cup performed by dominant hands of surgeons is more accurate than that performed by non-dominant sides.

## Background

Acetabular cup orientation including anteversion and inclination is a crucial factor for the functional outcome after total hip arthroplasty (THA) [1, 2]. Many factors including the surgical approach and pelvic movement that affect cup orientation have already been discussed [3, 4], but there were few articles about the influence of surgeon handedness. In Pennington et al.’s [5] study, they reported the influence of surgeon handedness on leg length inequality, acetabular inclination, and center of rotation.

However, it remains unknown whether surgeon handedness has an effect on acetabular anteversion and which cup placement performed by left or right hand could be more accurate. Orthopedic surgeons operate on both sides of patients’ hips in many musculoskeletal disorders that affect hips such as osteonecrosis, osteoarthritis, dysplasia, ankylosing spondylitis, and so on. Their operating position changes with different sides of patients when THA is being performed, particularly when patients are in lateral decubitus position with the posterolateral approach. Therefore, surgeon handedness may exert an influence on subject judgment of placing acetabular cup at a proper orientation.

During the procedure of performing right THA, orthopedic surgeons stand on the right side of the patients which will be comfortable and convenient to ream and implant acetabular component for a right-handed surgeon. However, when performing left THA, this situation will become complicated. This change of surgeons’ spatial position perhaps results in difference of bilateral acetabular orientation.

The primary purpose of this study was to investigate the significant difference of cup orientation (anteversion/inclination) in bilateral total hip arthroplasty (BTHA) performed by right-handed surgeons. The secondary purpose was to determine which cup placement could be fitter to the safe zone.

## Methods

A consecutive of series of 498 patients (996 hips) from January 2013 to December 2015 were retrospectively reviewed in our center and had a minimum follow-up of 6 months. These THAs were performed by three experienced orthopedic surgeons (surgeon A, B, and C) who had performed more than 300 THAs annually. In this study, these three surgeons performed all of the operations while standing on the same side of the operative hip intending to put the acetabular cup in their own “target zone.” A target zone refers to that where surgeons wanted to put the acetabular cup according to their experience and habits. All of these target zones were selected in the safe zone according to Lewinnek et al. [6]. In their study, they suggested a relatively safe range of cup orientation with an anteversion of 15° ± 10° and an inclination of 40° ± 10°. These three surgeons underwent assessments of the Edinburgh Handedness Inventory [7], and they were defined as right-handers. Press-fit acetabular components and ceramic insert (Ceramtec AG, Plochingen, Germany) were used. At the second day after THA, each patient had a standard anteroposterior (AP) pelvic radiography (focus-film distance: 1150 mm) in supine position following the hospital’s standard.

### Inclusion/exclusion criteria

Patients who met the following criteria were included in this study: (1) All procedures performed in lateral decubitus position with the posterolateral approach, (2) bilateral total hip arthroplasty completed by the same surgeon, (3) patients who underwent primary THA, (4) the same kind of prosthesis component used in BTHA, (5) bilateral hip with same stage of disease and bilateral acetabulum with similar bone mass, (6) patients without Crowe type-IV DDH, and (7) patients without deformity of the hip.

### Measurements of acetabular cup orientation

According to the definition of Murray [8], acetabular anteversion and inclination have been defined as radiographic, anatomic, and operative. In our study, radiographic definition was used to measure anteversion and inclination on AP pelvic radiograph within the coronal plane.

The anteversion angle and inclination of the acetabular cup were measured by an angular measurement tool (Orthoview Digital Planning Software) on an AP pelvic radiograph. This software is a validated tool as described by Restrepo [9]. In his study, he compared measurements of inclination and anteversion in 22 hips on pelvic CT scans with pelvic radiographs and found no significant difference between them. The cup angle was measured once by two independent observers who were blinded to each other’s result to check interobserver reliability.

**Fig. 1:**
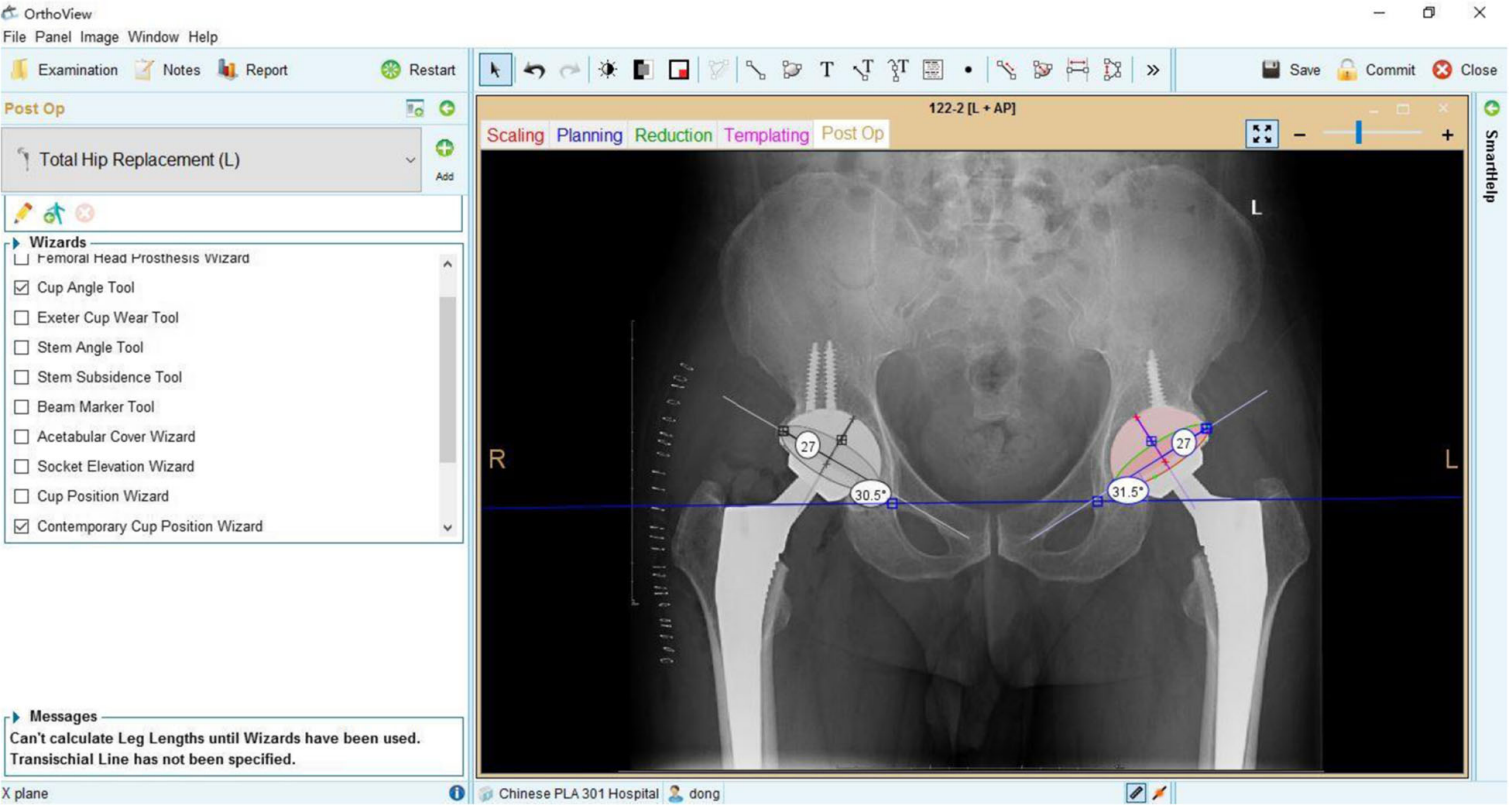
Method for measuring acetabular anteversion and inclination on an AP pelvic radiography using the Orthoview Digital Planning Software system

As a reference plane for inclination measurements, we used the inter-teardrop line in most of the patients [10, 11]. Vahdettin found that use of the inter-teardrop line as a pelvic landmark is preferential to that of the bi-ischial line because of its lower impact on the position of the pelvis [12]. However, in patients with developmental dysplasia of the hip (DDH), we used the bi-ischial line as the baseline because the teardrops tend to be difficult to identify on AP pelvic radiograph. The acetabular inclination was measured as the angle between a line connecting the most caudal points of pelvic teardrops or ischial tuberosities and the long acetabular axis. Acetabular anteversion was measured by drawing the ellipse of the acetabular cup’s opening rim followed by determination of the short and long axes of the ellipse (Fig. 1).

### Statistics

SPSS version 21 (IBM, New York, US) was used for statistics analysis. Mean acetabular cup anteversion and inclination between left and right component were compared using paired samples *t* test. The mean operative time of left and right THAs were compared using paired samples *t* test. The percentage of cup placement (anteversion, inclination, and combined) within the safe zone and target zone for each side hip and dislocation rates between two sides were compared using the chi-square test. The level of significance was set at *p* < 0.05. Interobserver reliabilities were assessed by the interclass correlation coefficient (ICC).

## Results

The cohort mean age of patients was 45.8 years, the mean body mass index (BMI) was 24.41 kg/m^2^, and most patients were male (61.6%). The main diagnosis was osteonecrosis of the femoral head (ONFH) (48.2%). The majority of acetabular implants were Betacup (Link, Germany) (66.3%) (Table 1). The mean operative time was 85.8 min in left THA and 83.8 min in right THA (*p* = 0.02), respectively. The rate of dislocation was 7.0% (35/498) in left THA and 3.2% (16/498) in right THA (*p* = 0.006). The Edinburgh inventory laterality quotients of surgeon A, B, and C were + 100, + 50 and + 70, respectively. The questionnaire results supported that they were right-handers as same as what they declared.

**Table 1:** Characteristics of patients and acetabular implants

Characteristic	Total *n* = 498
Gender *n* (%)	
Male	307 (61.6)
Female	191 (38.4)
Age (years), mean (SD), range	45.8 (12.8), 20–83
BMI (kg/m^2^), mean (SD), range	24.41 (3.95), 13.2–44.8
Diagnosis, *n* (%)	
Osteoarthritis	112 (22.5)
ONFH	240 (48.2)
DDH	24 (4.8)
AS	107 (21.5)
RA	15 (3.0)
Acetabular type, *n* (%)	
Betacup (Link, Germany)	330 (66.3)
Pinnacle (DePuy, USA)	97 (19.5)
Combicup (Link, Germany)	71 (14.2)

*n* number, *SD* standard deviation, *BMI* body mass index, *ONFH* osteonecrosis of the femoral head, *DDH* developmental dysplasia of the hip, *AS* ankylosing spondylitis, *RA*, rheumatoid arthritis

**Fig. 2:**
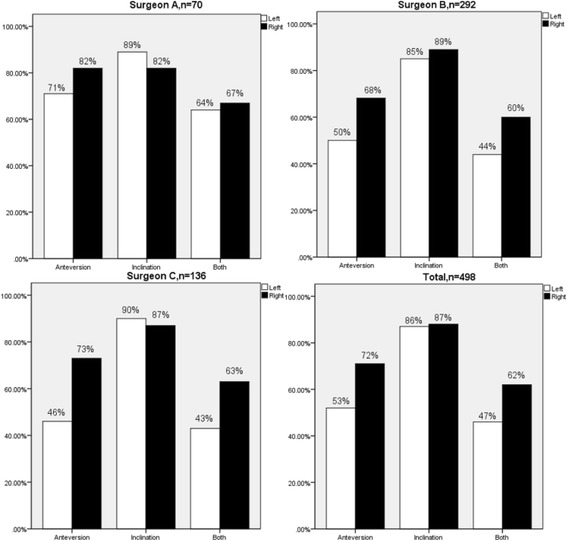
The percentage of safe zone placement for inclination, anteversion, and both for left and right THA in surgeon A, B, C, and all patients

In left THA, the mean cup placement for inclination was 37.50° (range 15.5–70, standard deviation [SD] 6.76); the mean cup placement for anteversion was 25.28° (range 6.5–45, SD 7.16). According to the criteria of Lewinnek et al., 433 (87%) from 498 cups were placed within the safe zone for inclination, and 259 (52%) from 498 cups were placed within the safe zone for anteversion. With regard to both inclination and anteversion, 230 (46%) from 498 cups were placed within the safe zone (Table 2) (Fig. 2).

**Table 2:** Data on cup orientation and safe zone in left THA and right THA

	Left THA (*n* = 498)	Right THA (*n* = 498)	*p* value
Mean inclination and anteversion			
Mean inclination (range, SD)	37.50 (15.5–70, 6.76)	38.59 (18–72.5, 6.84)	0.011*
Mean anteversion (range, SD)	25.28 (6.5–45, 7.16)	22.01 (7.5–41.5, 6.35)	< 0.001*
Safe zone placement			
Inclination	433/498 (87%)	436/498 (88%)	0.776
Anteversion	259/498 (52%)	356/498 (71%)	< 0.001*
Inclination and anteversion	230/498 (46%)	307/498 (62%)	< 0.001*

*n* number, *SD* standard deviation, *THA* total hip arthroplasty

*Has statistical significance

In right THA, the mean cup placement for inclination was 38.59° (range 18–72.5, SD 6.84), and the mean cup placement for anteversion was 22.01° (range 7.5–41.5, SD 6.35). According to the criteria of Lewinnek et al., 436 (88%) from 498 cups were placed within the safe zone for inclination, and 356 (71%) from 498 cups were placed within the safe zone for anteversion. With regard to both inclination and anteversion, 307 (62%) from 498 cups were placed within the safe zone (Table 2) (Fig. 2).

**Fig. 3:**
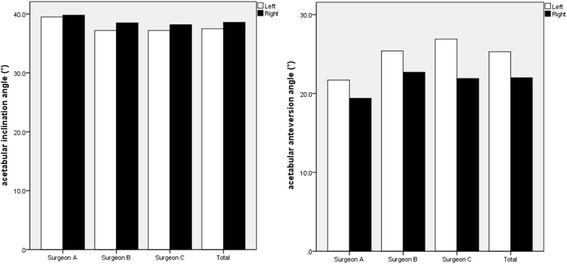
Mean acetabular inclination and anteversion for left and right THAs of these three surgeons

The mean difference was 1.08° (range − 47–26, SD 9.46) for inclination and 3.27° (range, − 17.5-24.5, SD7.37) for anteversion between left and right THA. There was a significant difference of mean inclination (*p* = 0.011) and mean anteversion (*p* < 0.001) between left and right THA (Fig. 3). The chi-square test revealed no significant difference in the proportion of safe zone for cup inclination (*p* = 0.776) but significant difference for cup anteversion (*p* < 0.001), and both inclination and anteversion (*p* < 0.001). There was no significant difference in the percentage of target zone for cup inclination, anteversion, and combined between left THA and right THA for three surgeons (Table 3).

**Table 3:** Target zone of placing cup for three surgeons and the percentage of target zone placement for inclination, anteversion, and both for left and right THA in three surgeons

		Target zone (°)	Left THA	Right THA	*p* value
Surgeon A (*n* = 70)	Inclination Anteversion Both	35–45 15–25	39/70 (56%) 40/70 (57%) 24/70 (34%)	37/70 (53%) 40/70 (57%) 20/70 (29%)	0.734 1.0 0.466
Surgeon B (*n* = 292)	Inclination Anteversion Both	35–40 20–25	99/292 (34%) 79/292 (27%) 31/292 (11%)	93/292 (32%) 92/292 (32%) 32/292 (11%)	0.597 0.237 0.894
Surgeon C (*n* = 136)	Inclination Anteversion Both	40–50 20–25	50/136 (37%) 46/136 (34%) 17/136 (13%)	49/136 (36%) 41/136 (30%) 17/136 (13%)	0.900 0.516 1.0

*n* number, *THA* total hip arthroplasty

Analysis of the interclass correlation coefficient showed that measurements between two observers were reliable. In left THA, the interobserver reliability was 0.97 (95% confidence interval [CI] 0.97; 0.98) for anteversion and 0.97 (95% CI 0.96; 0.97) for inclination, respectively. In right THA, the interobserver reliability was 0.96 (95% CI 0.95; 0.97) for anteversion and 0.97 (95% CI 9.97; 0.98) for inclination, respectively. Thus, we chose one measurement to analyze.

## Discussion

Considering that the position of the acetabular cup plays an influential role in THA outcome, orthopedic surgeons should attempt to exclude multi-adverse factors but concentrate on performing a successful cup placement. Our study suggests that there was a significant difference for either inclination or anteversion between left THA and right THA performed by same right-handed surgeon. In Pennington et al.’s study [5], they found that the difference for inclination was 3° between THAs performed by the dominant and non-dominant sides of surgeons, which was higher than that in our study. That is to say, surgeon handedness has an influence on both cup anteversion and inclination. Furthermore, we compared the percentage of cup placement within the safe zone for each side hip and found that the mean cup placement for anteversion in right THA was lower than in left THA; however, the percentage of safe zone for cup anteversion was higher in right THA than in left THA. Our study also suggests that right-handed surgeons put the acetabular cup more ideal and accurate in right THA than in left THA. Plenty of studies had reported that cup anteversion in THA is a key factor that relates to dislocation of hip [13–15]. In our study, we found out that the dislocation rate in left THA was significantly higher than right THA (*p* = 0.006).

There were few papers previously that addressed the effect of surgeon handedness on surgical outcome. Pennington et al. [5] firstly reported a study including a series of 160 THAs, equal numbers of left and right THAs which were performed averagely by two left-handed and two right-handed surgeons. Leg length inequality, acetabular inclination and center of rotation were measured on postoperative AP X-ray. The result showed that surgeon handedness did appear to influence the acetabular component position. Moloney et al. [16] compared the preoperative and postoperative X-rays of 244 basic cervical or intertrochanteric hip fractures which were fixed by sliding hip screws. They concluded that malposition of the 12 failures occurred more commonly on the left side when the surgeon is right-handed. In Mehta and Lotke’ s [17] study, they considered a series of 728 primary TKAs which were performed by a right-handed surgeon standing on the side of the operative extremity. Function and pain scores 1 year after surgery showed that handedness could play a role in TKA outcomes.

**Fig. 4:**
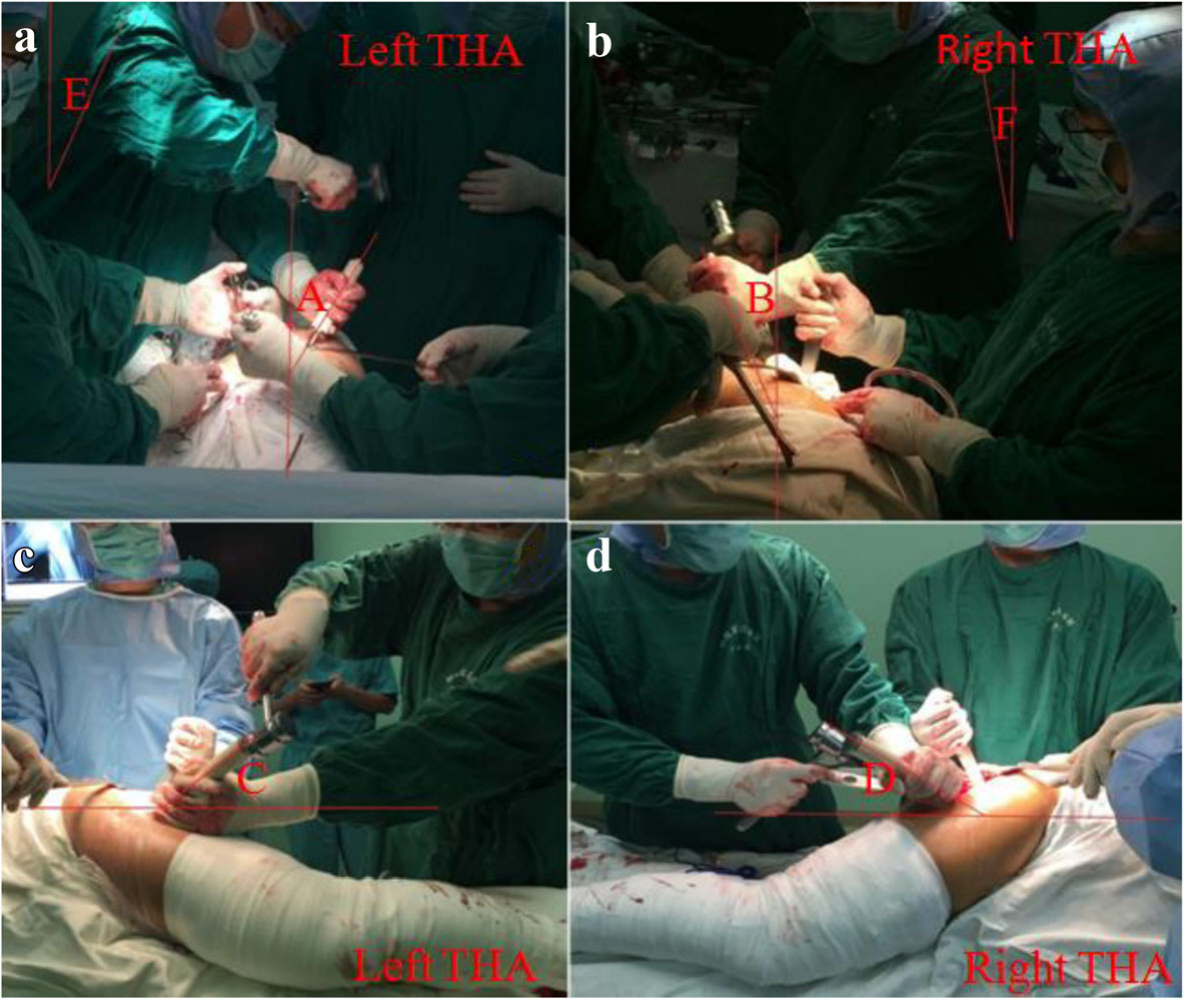
**a**–**d** Spatial position of the surgeon during left and right THA. A and C are anteversion and inclination of left acetabular cup, B and D are anteversion and inclination of right acetabular cup, E and F are title angles of a surgeon body in left and right THA

We have not yet found the reasons responsible for this observed difference in bilateral cup orientation, but we have posed a possible hypothesis. In our study, all patients who underwent THA were placed in lateral decubitus position. During left THA, the surgeon’s right hand with a hammer needs to operate above the left hand when implanting acetabular component. In this case, the left hand of a surgeon is usually located lower than in the right THA to get enough operating space for the right hand (Fig. 4c). However, the operating space of the right hand is less likely to be affected in the right THA. In this case, the position of the left hand is more suitable with enough operating space for the right hand to implant the acetabular component during right THA (Fig. 4d). This difference of spatial position of two hands in left THAs resulted in lower inclination angles of cup components. With respect to cup anteversion, we presume that the tilt of a right-handed surgeon’s body contributes to the larger cup anteversion in left THA (Fig. 4a, b). In addition, we also found significant differences of operative time between the two sides. One explanation may be that it is more comfortable and convenient for a right-handed surgeon to operate right THA than on the opposite side; thus, he can finish the right operation faster. Further investigation to elucidate the reason is warranted.

We acknowledge several limitations of our study. At first, the three surgeons included in this study are all right-handers, which probably results in one-sidedness of the result. This study presents early data, and we will also recruit left-handed surgeons to our study in the following research. Secondly, in order to exclude possible effect of surgical position and approach on the result, all of enrolled patients were performed in the lateral position using posterolateral approach. Is there still difference for cup orientation between two sides if patients undergo THA in the supine position? In theory, the anterolateral approach in the supine position can provide adequate visualization of the acetabulum and operating space for surgeons. Otherwise, in Coleman et al.’ s study [18], the anterolateral approach was used in 91 hips, the transtrochanteric approach in 136, and the posterior approach in 42. They found that acetabular cup orientation showed no significant differences between the three surgical approaches. Rothman [19] thought that the operative approach affects the planned orientation of the acetabulum, primarily anteversion. Further research is needed. Lastly, the major limitation of this research could be the single-center study design. Despite all this, the observed differences of acetabular component orientation are firstly reported, especially with regard to cup anteversion. Additionally, this research sample is large enough. Further research would be conducted to eliminate the above limitations.

## Conclusion

Our data demonstrates that surgeon handedness is likely to be a contributing factor that affects cup inclination and anteversion in performing a THA and the placement of cups performed by the dominant hands of surgeons is more accurate than that performed by the non-dominant sides. This study is clinically significant; thus, orthopedic surgeons should take this potential problem into consideration and take precautions to prevent diminished results, particularly when preparing acetabulum on the non-dominant side of the body.

## Abbreviations

AP: Anteroposterior
AS: Ankylosing spondylitis
BMI: Body mass index
BTHA: Bilateral total hip arthroplasty
CI: Confidence interval
DDH: Developmental dysplasia of the hip
ICC: Interclass correlation coefficient
ONFH: Osteonecrosis of the femoral head
RA: Rheumatoid arthritis
SD: Standard deviation
THA: Total hip arthroplasty

## References

[1] Hube R, Dienst M, von Roth P (2014). Complications after minimally invasive total hip arthroplasty. Orthopade.

[2] Harrison CL, Thomson AI, Cutts S (2014). Research synthesis of recommended acetabular cup orientations for total hip arthroplasty. J Arthroplast.

[3] Grammatopoulos G, Pandit HG, da Assuncao R (2014). The relationship between operative and radiographic acetabular component orientation: which factors influence resultant cup orientation?. Bone Joint J.

[4] Asayama I, Akiyoshi Y, Naito M (2004). Intraoperative pelvic motion in total hip arthroplasty. J Arthroplast.

[5] Pennington N, Redmond A, Stewart T (2014). The impact of surgeon handedness in total hip replacement. Ann R Coll Surg Engl.

[6] Lewinnek GE, Lewis JL, Tarr R (1978). Dislocations after total hip-replacement arthroplasties. J Bone Joint Surg Am.

[7] Oldfield RC (1971). The assessment and analysis of handedness: the Edinburgh inventory. Neuropsychologia.

[8] Murray DW (1993). The definition and measurement of acetabular orientation. J Bone Joint Surg Br.

[9] Restrepo C, Parvizi J, Kurtz SM (2008). The noisy ceramic hip: is component malpositioning the cause?. J Arthroplast.

[10] Todkar M (2008). Obesity does not necessarily affect the accuracy of acetabular cup implantation in total hip replacement. Acta Orthop Belg.

[11] Wan Z, Malik A, Jaramaz B (2009). Imaging and navigation measurement of acetabular component position in THA. Clin Orthop Relat Res.

[12] Bayraktar V, Weber M, von Kunow F, et al. Accuracy of measuring acetabular cup position after total hip arthroplasty: comparison between a radiographic planning software and three-dimensional computed tomography. Int Orthop. 2016; 10.1007/s00264-016-3240-1 27277948

[13] Abdel MP, von Roth P, Jennings MT (2016). What safe zone? The vast majority of dislocated THAs are within the Lewinnek safe zone for acetabular component position. Clin Orthop Relat Res.

[14] Elkins JM, Callaghan JJ, Brown TD (2015). The 2014 Frank Stinchfield award: the ‘landing zone’ for wear and stability in total hip arthroplasty is smaller than we thought: a computational analysis. Clin Orthop Relat Res.

[15] Fujishiro T, Hiranaka T, Hashimoto S (2016). The effect of acetabular and femoral component version on dislocation in primary total hip arthroplasty. Int Orthop.

[16] Moloney D, Bishay M, Ivory J (1994). Failure of the sliding hip screw in the treatment of femoral neck fractures: ‘left-handed surgeons for left-sided hips’. Injury.

[17] Mehta S, Lotke PA (2007). Impact of surgeon handedness and laterality on outcomes of total knee arthroplasties: should right-handed surgeons do only right TKAs?. Am J Orthop (Belle Mead NJ).

[18] Vicar AJ, Coleman CR (1984). A comparison of the anterolateral, transtrochanteric, and posterior surgical approaches in primary total hip arthroplasty. Clin Orthop Relat Res.

[19] Austin MS, Rothman RH (2009). Acetabular orientation: anterolateral approach in the supine position. Clin Orthop Relat Res.

